# Accurate Decoding of Imagined and Heard Melodies

**DOI:** 10.3389/fnins.2021.673401

**Published:** 2021-08-05

**Authors:** Giovanni M. Di Liberto, Guilhem Marion, Shihab A. Shamma

**Affiliations:** ^1^Laboratoire des Systèmes Perceptifs, CNRS, Paris, France; ^2^Ecole Normale Supérieure, PSL University, Paris, France; ^3^Department of Mechanical, Manufacturing and Biomedical Engineering, Trinity Centre for Biomedical Engineering, Trinity College, Trinity Institute of Neuroscience, The University of Dublin, Dublin, Ireland; ^4^Centre for Biomedical Engineering, School of Electrical and Electronic Engineering and UCD University College Dublin, Dublin, Ireland; ^5^Institute for Systems Research, Electrical and Computer Engineering, University of Maryland, College Park, College Park, MD, United States

**Keywords:** EEG, cortical, TRF, neural tracking, pitch, music, signal processing, decoding

## Abstract

Music perception requires the human brain to process a variety of acoustic and music-related properties. Recent research used encoding models to tease apart and study the various cortical contributors to music perception. To do so, such approaches study temporal response functions that summarise the neural activity over several minutes of data. Here we tested the possibility of assessing the neural processing of individual musical units (bars) with electroencephalography (EEG). We devised a decoding methodology based on a maximum correlation metric across EEG segments (*maxCorr*) and used it to decode melodies from EEG based on an experiment where professional musicians listened and imagined four Bach melodies multiple times. We demonstrate here that accurate decoding of melodies in single-subjects and at the level of individual musical units is possible, both from EEG signals recorded during listening and imagination. Furthermore, we find that greater decoding accuracies are measured for the *maxCorr* method than for an envelope reconstruction approach based on backward temporal response functions (*bTRF*_*env*_). These results indicate that low-frequency neural signals encode information beyond note timing, especially with respect to low-frequency cortical signals below 1 Hz, which are shown to encode pitch-related information. Along with the theoretical implications of these results, we discuss the potential applications of this decoding methodology in the context of novel brain-computer interface solutions.

## Introduction

In our everyday life, our brain examines sounds by extracting various types of auditory features. In music, one such feature is the melody, which is a sequence of pitches set to a particular rhythm in which the individual tones are processed in terms of multiple structured relationships (Patel, 2003). The melody is generally one important part of a song that we remember and we enjoy humming or whistling. However, the neural processes leading to the extraction of melodies from complex auditory stimuli remain unclear. Recent work has provided new insights into this process by studying the neural activity recorded with electroencephalography (EEG) during music listening tasks (Carrus et al., 2013; Omigie et al., 2013; Di Liberto et al., 2020). That work used encoding models and found that neural signals encode both the acoustic properties of music (timing and pitch of a note) and the expectation of musical notes according to the preceding proximal context and the musical background of the listener (Di Liberto et al., 2020).

The ability to measure this multifaceted neural encoding of melodies with non-invasive brain recordings provides us with new opportunities to unveil the neural encoding of melodies and its precise role in music perception. The encoding modelling framework constitutes an effective solution to study the neural processing of complex sounds, such as melodies, by teasing apart its various cortical contributors (Koelsch, 2011; Brodbeck et al., 2018; Obleser and Kayser, 2019; Di Liberto et al., 2021b). While that work informed us on which properties of music are encoded in cortical signals measured, hence contributing to our understanding of how the human brain processes melodies, the present study investigated the inverse question: can we use such cortical signals to identify the corresponding melodies that were either listened to or imagined? A successful classifier can then be studied to determine which particular melodic properties are encoded in the neural signal and contribute to the decoding. Furthermore, the development of such a classifier for the case of music imagery could directly translate into new brain-computer interfaces (BCIs).

Recent work demonstrated that EEG signals recorded during music listening (Di Liberto et al., 2020) and imagery (Marion et al., 2021) encode both the sound envelope and higher-order neural activity reflecting melodic predictions. Here we devised a framework to capture this rich spectrum of signals including both low- and higher-level neural processes, with the goal of accurately decoding melodies from EEG. We present a re-analysis of a publicly available dataset previously published by our team (Di Liberto et al., 2021a; Marion et al., 2021). EEG signals were recorded as participants listened or imagined Bach melodies. The start of each musical measure was indicated to the participants with a vibro-tactile metronome placed on their ankle, which provided an important synchronisation signal for the imagery task, but that was also presented in the listening condition for consistency and was identical across melodies. The study tested for the possibility that listened and imagined Bach melodies could be precisely decoded from EEG. Our classification approach consisted of partitioning the EEG signal into segments of a given length (1, 2, 4, or 8 music bars), and then assigning each EEG segments with the note sequence corresponding to the most similar EEG segment in the dataset. The decoding quality was then assessed both in terms of note timing and pitch value information. Crucially, we used melodies with the same tempo to ensure that the decoding was driven by the neural encoding of melodies rather than differences in tempo.

## Materials and Methods

### Data Acquisition and Experimental Paradigm

Twenty-one healthy individuals (6 female, aged between 17 and 35, median = 25) participated in the EEG experiment, which was conducted as part of a previous study (Di Liberto et al., 2021a; Marion et al., 2021). All participants were highly trained musicians with music degree. Ten of them were professional musicians. The other eleven participants were studying to become professional musicians at the CNSM (six of them) and the CRR (five of them) Paris institutes. Eighteen participants had strong expertise in at least one music instruments (piano, guitar, saxophone, violin, percussions, cello, clarinet, accordion, double bass, flute), two were singers, and one was an expert in music theory. Each subject reported no history of hearing impairment or neurological disorder, provided written informed consent, and was paid for their participation. The study was undertaken in accordance with the Declaration of Helsinki and was approved by the CERES Committee of Paris Descartes University (CERES 2013-11). The experiment was carried out in a single session for each participant. EEG data were recorded from 64 electrode positions and digitised at 2,048 Hz using a BioSemi Active Two system. Three additional electrodes were placed on the upper midline of the neck, the jaw, and the right wrist to control for motor movements of the tongue, masseter muscle, and forearm fingers extensors, respectively. Audio stimuli were presented at a sampling rate of 44,100 Hz using a Genelec 8010-10w loud-speaker and custom Python code. Testing was carried out at École Normale Supérieure, in a dimmed room. Participants were instructed to minimise motor activities while performing the task.

The experiment consisted of 88 trials in which participants were asked to either listen or perform mental imagery of ∼35 s melodies from a corpus of Bach chorales (see section “Stimuli and Procedure”). Participants were asked to read the music scores placed at the centre of the desk during both listening and imagery conditions. A tactile metronome (Peterson Body Beat Vibe Clip) marking the start of 100 bpm bars (each 2.4 s) was placed on the left ankle of all participants to allow them to perform the mental imagery task with high temporal precision. A constant lag of 35 ms was determined during the pilot experiments based on the subjective report by the participants, who reported that the metronome with lag 0 ms was not in sync with the music. That correction was applied for all participants with the same lag value. Before the experiment, musical imagery skills (or audiation skills) were assessed for every subject with “The Advanced Measures of Music Audiation” test^1^ (AMMA).

### Stimuli and Procedure

Four melodies were selected from a monophonic MIDI corpus of Bach chorales (BWV 349, BWV 291, BWV354, BWV 271). Each melody was repeated 11 times per condition and the order of melodies (four melodies) and conditions (listening and imagery) was randomised. All chorales use similar compositional principles: the composer takes a melody from a Lutheran hymn (*cantus firmus*) and harmonises three lower parts (alto, tenor, and bass) accompanying the initial melody on soprano. The monophonic version of those melodies consist of the *cantifirmi*. Original keys were used. The four melodies are based on a common grammatical structure and show very similar melodic and rhythmic patterns. The audio stimuli were synthesised using a Fender Rhodes simulation software (Neo-Soul Keys) with 100 bpm, each corresponding to the start of a bar (every 2.4 s).

### EEG Data Preprocessing

Neural data were analysed offline using MATLAB software (The Mathworks Inc.). EEG signals were digitally filtered between 0.1 and 30 Hz using a Butterworth zero-phase filter (low- and high-pass filters both with order 2 and implemented with the function *filtfilt*), and down-sampled to 64 Hz. The analyses were also conducted on EEG data filtered between 1 and 30 Hz (Butterworth zero-phase filters with order 2) to assess the impact of low-frequency EEG < 1 Hz, which was previously suggested to encode a relevant portion of imagery EEG signals (Marion et al., 2021). EEG channels with a variance exceeding three times that of the surrounding ones were replaced by an estimate calculated using spherical spline interpolation. Channels were then re-referenced to the average of the 64 channels.

### Multiway Canonical Correlation Analysis (MCCA)

A multiway canonical correlation analysis (MCCA) was performed to combine EEG data across subjects to improve the SNR. MCCA is an extension of canonical correlation analysis (CCA; Hotelling, 1936) to the case of multiple (>2) datasets. Given N multichannel datasets Y_*i*_ with size T × J_*i*_, 1 ≤ i ≤ N (time × channels), MCCA finds a linear transform W_*i*_ (sizes J_*i*_ × J_0_, where J_0_ < min (J_*i*_)_1≤ i≤ N_) that, when applied to the corresponding data matrices, aligns them to common coordinates and reveals shared patterns (de Cheveigné et al., 2019). These patterns can be derived by summing the transformed data matrices: $\text{Y}=\sum_{\text{i}=1}^{\text{N}}{\text{Y}}_{\text{i}}\,{\text{W}}_{\text{i}}$. The columns of the matrix Y, which are mutually orthogonal, are referred to as summary components (SC). The first components are signals that most strongly reflect the shared information across the several input datasets, thus minimising subject-specific and channel-specific noise. Here, these datasets are EEG responses to the same task for 21 subjects. The present study used the implementation discussed by de Cheveigné et al. (2018; Matlab implementation available at http://audition.ens.fr/adc/NoiseTools/).

This technique allows the extraction of a “consensus signal” that is shared across subjects and has higher SNR than any individual subject signal. This methodology is a better solution than averaging data across subjects which, in absence of appropriate co-registration, can lead to loss of information because of topographical discrepancies. MCCA accommodates such discrepancies without the need for co-registration. Under the assumption that the EEG responses to music and music imagery share a similar time-course within a homogeneous group of young adults, the MCCA procedure allows us to extract such common cortical signals from other, more variable aspects of the EEG signals, such as subject-specific noise. For this reason, our analysis focuses on the first N_*SC*_ summary components, which we can consider as spanning the most reliable EEG response to music and music imagery. Because the resulting signals are then treated as a virtual best subject and compared with the results on individual participants, N_*SC*_ was set to 64 so that such a virtual subject would have the number of signals of the single participants in the original EEG dataset. This analysis was conducted on the 0.1–30 Hz and 1–30 Hz EEG datasets separately. Note that the trials have to be sorted to build the MCCA models, as different trial order was applied to different participants.

### Joint Decorrelation Analysis (JD)

The EEG signal contains responses driven by the stimulus as well as stimulus-irrelevant neural activity. By assuming that the stimulus-driven response is consistent over repetitions of a same melody, we decomposed the EEG signal using joint decorrelation analysis (JD) (de Cheveigné and Simon, 2008; de Cheveigné and Parra, 2014), a component analysis method that extracts neural activity consistent across repetitions. Specifically, JD maps the multivariate EEG recording into a space where each component is determined by maximising its trial-to-trial reliability, measured by the correlation between the responses to the same stimulus in different trials. The first JD component contains the most consistent neural response across repetitions. Here, we selected the first *M* components, where *M* was set so that the selected components would capture 50% of the variance (which generally corresponded to less than 10 out of 64 components). Note that this procedure was run separately for each participant, including the virtual MCCA participant. Components with topographical maps consistent with what would be expected for eye-movements and blinks have been removed via semi-supervised inspection (automatic identification of such components via custom code and manual inspection).

### Sound Envelope Reconstruction Analysis (bTRF)

Lagged linear regression models were fit to relate the music sound envelope and the corresponding EEG signal. Models describing the forward mapping from stimulus features to EEG signal can be referred to as temporal response functions (TRF), as their kernel (i.e., regression weights) estimates the neural impulse response (Lalor et al., 2009; Ding et al., 2014). The same method can also be used to describe the inverse mapping from the multivariate EEG space to the univariate stimulus feature space (backward model). Here, we used an implementation of such backward modelling approach based on a regularised lagged linear regression (Crosse et al., 2016) to map the EEG signal to the sound envelope of music that was either presented to or imagined by the participants. The present paper refers to this decoding methodology as backward TRF (bTRF). Leave-one-out cross-validation (across the 44 trials for each condition) was used to assess how well the decoding models could reconstruct sound envelopes for portions of the EEG data that was not included in the model fit, thus controlling for overfitting. Note that only one music segment was selected for each iteration, so the remaining dataset included trials with the same stimulus of the left-out trial (see section “Maximum Correlation Music Decoding”). The quality of a reconstruction was quantified by calculating Pearson’s correlation between the actual and the reconstructed signals.

The interaction between stimulus and recorded brain responses is not instantaneous, in fact a sound stimulus at time *t*_0_ affects the brain signals for a certain time-window (*t*_1_, *t*_1_+*t*_*win*_), with *t*_1_ ≥ 0 and *t*_*win*_ > 0. The linear decoding model takes this into account by including multiple time-lags between stimulus and neural signal, providing us with model weights that include both space (scalp topographies) and time (music-EEG latencies) dimensions. A time-lag window of 0–350 ms was selected as such latencies were expected to capture the relevant TRF response based on previous work (e.g., Freitas et al., 2018; Jagiello et al., 2019; Di Liberto et al., 2020, 2021a; Marion et al., 2021).

In order to decode the melody, stimulus and EEG data were partitioned into segments with a given fixed length. For each segments, Pearson’s correlations were calculated between the reconstructed envelope and the sound envelope of each original stimulus. The musical piece leading to the highest correlation was selected as the classification result for the particular EEG segment. This classification procedure (*bTRF*_*env*_) was run for segments with increasing duration (2.4, 4.8, 9.6, 19.2 s), which was always a multiple of the duration of a music bar (2.4 s).

One strength of this decoding approach is that it operates in the sound envelope domain, where the EEG signal was projected while reducing the impact of unrelated neural activity and EEG noise. However, the sound envelope encodes only note timing and loudness (however, all notes had the same loudness in this study), thus it cannot distinguish segments of music with the same timing. While this is not a problem when considering long segments of music or entire songs, it constitutes a limitation when decoding short segments of music.

### Maximum Correlation Music Decoding (maxCorr)

**FIGURE 1:**
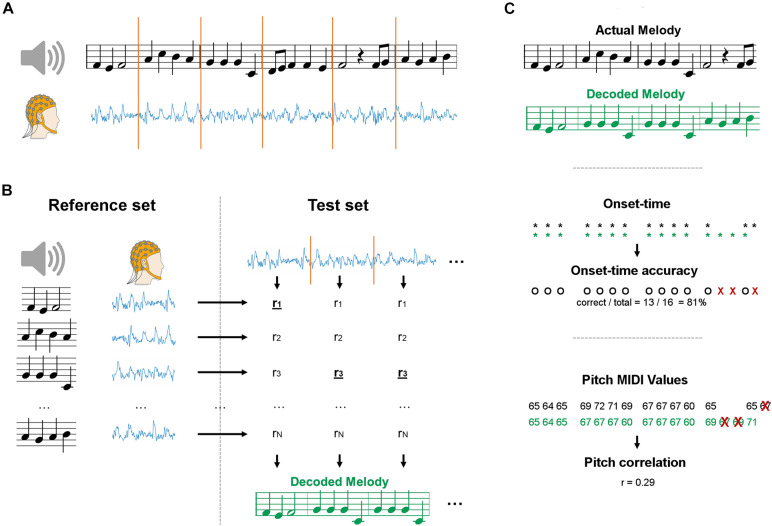
Schematics of the maxCorr decoding approach. **(A)** EEG data was recorded as participants either imagined or listened to monophonic music. Signals were segmented into a reference and test datasets. **(B)** EEG data from each segment was compared with the EEG segments in the reference set with cross-validation. The melody corresponding to the EEG segment with highest similarity was selected. The procedure was repeated for various segment sizes, always multiples of the size of a music bar. **(C)** The quality of the melody decoding was assessed by means of two distinct metrics. The first evaluation metric measured the accuracy in detecting the onset-time of the notes (black: actual melody; green: decoded melody). The second evaluation metric measured the correlation of the pitch values between decoded and actual melodies. The correlation was evaluated on the notes whose temporal onset was correctly identified (all other notes were discarded).

A maximum correlation classification method was devised to perform melody decoding in the EEG domain, without constraining the classification procedure to any predefined stimulus feature (e.g., sound envelope). In doing so, we expected the resulting decoding to be more accurate than bTRF_*env*_, which focuses on timing information only. Data were segmented and grouped into *reference* and *test* sets for a given subject (Figure 1A). Leave-one-outcross-validation was applied, meaning that a single segment was assigned to the *test set* and all other segments constituted the *reference set*. The decoding was performed on each *test set* by selecting the music segment in the *reference set* with most similar EEG, based on a EEG-EEG Pearson’s correlation metric (Figure 1B). We calculated the EEG-EEG Pearson’s correlation for each JD component, and then performed an average of those correlation scores multiplied by a weighting vector *w*, which has as many elements as the number of selected JD components. The weighting vector was calculated by deriving the root mean squared for each of the selected components over the entire duration of the experiment for a given subject and condition. As such, more importance was given to the correlation scores for JD components that had larger variance over the entire duration of the experiment. The *reference* and *test sets* of melody segments were built by partitioning melodies into chunks with a fixed length *w* of 1, 2, 4, or 8 bars (corresponding to 2.4, 4.8, 9.6, and 19.2 s). Note that, by partitioning we refer to a chunking based on a moving window with an equal window size (*w*) and step. The reference set contained the same segments in the test set, but was augmented by setting the step to 1 bar for all window sizes. The entire procedure was repeated for each subject and for the various segment sizes, always multiples of the length of a music bar. Note that longer segments would lead to smaller reference sets (even though we used an epoching step of 1, less segments can be derived at the end of each trial). Indeed, reference sets of different size may lead to potentially largely different baselines. To avoid this issue, we have resampled the reference set to 96 segments at each iteration, where 96 was the size of the smallest reference set (for the longest segment length).

### Evaluation Metrics

**FIGURE 2:**
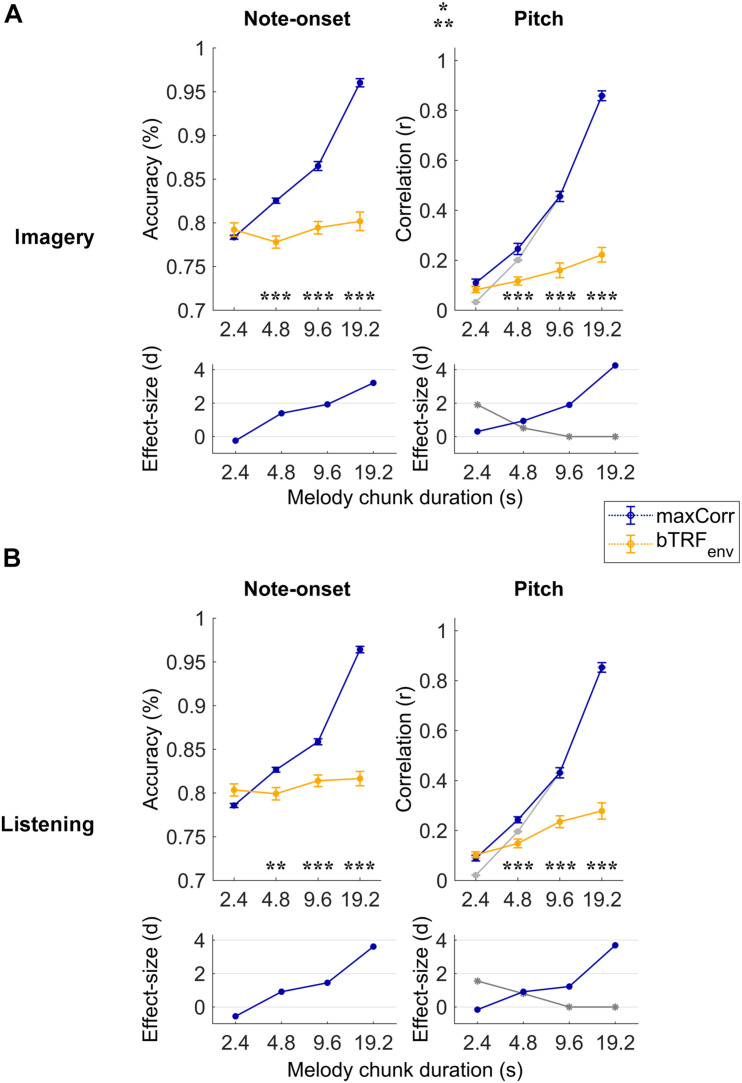
Robust note and pitch decoding from EEG. Melody decoding scores obtained with maxCorr from EEG data filtered between 0.1 and 30 Hz are compared with scores obtained when using backward envelope TRFs (bTRF_*env*_) for the imagery **(A)** and listening **(B)** conditions. Decoding accuracies are reported for note timing (left) and decoding correlation values for note pitch values (right). As expected, decoding scores were higher for maxCorr than bTRF_*env*_ (three-way repeated measures ANOVA *p* < 0.001, *post hoc* Tukey’s HSD; *p < 0.05, **p < 0.01, ***p < 0.001). Grey lines indicate the pitch decoding correlation scores with the maxCorr method when pitch information was shuffled among segments with identical timing. Scores larger than this baseline indicate that the decoding is partially driven by pitch-related EEG information. Bottom panels indicate the effect size (Cohen’s d) of the comparison between maxCorr and bTRF_*env*_ (blue solid lines) and between maxCorr and the maxCorr baseline after shuffling the pitch values (grey solid lines). Note that longer segments would lead to smaller reference sets, thus potentially affecting the decoding baseline. This potential confound was solved by randomly resample the reference set to the size of the smallest set across all segment durations (see section “Materials and Methods”). Supplementary Figure 1 shows the results of this analysis for EEG filtered in the frequency band 1–30 Hz.

Two distinct evaluation metrics were devised to compare the decoding results for *bTRF*_*env*_ and *maxCorr* (Figure 1C). The first metric evaluated the accuracy in the decoding of *note-onsets*. Such a value was determined as the ratio between the number of correct identifications of note vs. silence and the total number of possible note positions (obtained by considering all segments in the reference set). The second metric assessed the Spearman’s correlation between the actual and decoded *note pitch* values (using a Pearson’s correlation did not change the results). Note that the identified music segments sometimes have different note timing than the actual segments. In those cases, we calculated the Spearman’s correlation based on the pitch values for notes that were present in both the reconstructed and actual music segments. Furthermore, because the reference set was limited to the four Bach pieces presented in the experiment, some segments could have been precisely identified even just based on timing information, a consideration that becomes more relevant for longer segments, where the timing of a segment is more likely to be unique (in the given stimulus set). As such, we conducted a control analysis where the pitch decoding metric was evaluated based on timing only and tested whether the main decoding result was above that baseline, thus indicating that the decoding is driven by more than timing information. Such a baseline was derived by shuffling segments with identical timing (but a potentially different sequence of pitch values) in each reference set before running the decoding (grey lines in Figure 2). This baseline allowed us to assess whether EEG encoded pitch-related information beyond the note timing.

### Statistical Analysis

Three-way repeated measures ANOVAs were performed to assess the effect of segment length (2.4, 4.8, 9.6, 19.2 s), decoding method (*bTRF*_*env*_ and *maxCorr*), and condition (imagery and listening). Separate tests were run for each evaluation metric (note-onset and pitch). Greenhouse-Geisser corrections were made if Mauchly’s test of sphericity was not met. All *post hoc* model comparisons were performed using the Tukey’s HSD test, which performs statistical tests while accounting for multiple comparisons. Effects within individual participants were assessed by means of permutation tests with *N* = 100 shuffles.

## Results

Melodies were decoded with the *maxCorr* method on individual subjects by considering progressively longer segments of EEG data (melody segment duration). Longer segments contain more information, thus they were expected to lead to higher decoding scores. The decoding scores with this procedure were then compared with the results when maxCorr was based on the envelope reconstructions obtained with *bTRF*_*env*_. Figure 2 depicts this quantitative comparison for each experimental condition (imagery vs. listening) and decoding quality metric (note-onset accuracy vs. pitch sequence correlation) when considering the broadband EEG signal (0.1–30 Hz). A three-way repeated measures ANOVA on the note-onset decoding accuracy metric indicated significant effects of: method [maxCorr vs. bTRF_*env*_; *F*(1, 20) = 80.6, *p* = 1.9 × 10^–8^]; segment duration [*F*(3, 20) = 431.2, *p* = 2.6 × 10^–21^]; and condition [imagery vs. listening; *F*(1, 20) = 6.0, *p* = 0.02]. Significant interactions were found for decoding method with segment duration [*F*(3, 60) = 295.7, *p* = 1.6 × 10^–20^] and with condition [*F*(1, 20) = 14.0, *p* = 1.3 × 10^–3^]. No significant interaction was measured between segment duration and condition [*F*(3, 60) = 0.48, *p* = 0.64]. A three-way repeated measures ANOVA on the pitch decoding correlation metric indicated significant effects of method [*F*(1, 20) = 142.3, *p* = 1.5 × 10^–10^] and segment duration [*F*(3, 20) = 524.2, *p* = 5.1 × 10^–34^], but no significant effect of condition was found [*F*(1, 20) = 1.8, *p* = 0.19]. As for the note-onset metric, significant interactions were shown for the pitch metric between decoding method and segment duration [*F*(3, 60) = 294.6, *p* = 1.6 × 10^–30^] and between method and condition [*F*(1, 20) = 8.2, *p* = 9.4 × 10^–3^]. No significant interaction was measured between segment duration and condition [*F*(3, 60) = 0.6, *p* = 0.55]. *Post hoc* Tukey’s HSD tests indicated that maxCorr produces overall better decoding scores than bTRF_*env*_ across all conditions and decoding metrics, with this gain being larger for increasing segment duration with similar decoding scores for imagery and listening. This result is in line with our hypothesis that the EEG data contains information relevant to decoding melodies that cannot be captured with envelope based linear decoding models.

The use of two distinct decoding metrics (note-onset classification accuracy and pitch correlation) informed us on the quality of the decoded melody from both the timing and pitch perspectives. Note that the range of possible music bars was confined to the particular musical pieces used in the EEG experiment. As such, the accurate decoding of note-onsets from EEG could lead to the identification of the exact stimulus segment, thus to the precise identification of pitch progressions. To assess whether pitch-related EEG signals contributed to the decoding of melodies, the procedure was repeated after shuffling the pitch among segments with identical timing, providing us with the pitch decoding correlation based solely on EEG signals reflecting note-onsets (grey lines in Figure 2). The pitch decoding correlation with maxCorr was significantly larger than that baseline for segment durations of 2.4 and 4.8 s in both conditions, indicating a significant contribution of pitch-related EEG information to the decoding (Tukey’s HSD, *p* < 0.05). As expected, such a contribution did not emerge for longer segment durations, which was an expected outcome because timing information is sufficient to uniquely characterise long segments of music.

Similar but overall weaker effects were measured when considering the EEG signal filtered in the band 1–30 Hz (Supplementary Figure 1). A three-way repeated measures ANOVA on the note-onset decoding accuracy metric indicated significant effects of: method [maxCorr vs. bTRF_*env*_; *F*(1, 20) = 5.8, *p* = 0.03]; segment duration [*F*(3, 20) = 89.4, *p* = 5.3 × 10^–11^]; and condition [imagery vs. listening; *F*(1, 20) = 74.0, *p* = 3.7 × 10^–8^]. Significant interactions were found for decoding method with segment duration [*F*(3, 60) = 177.3, *p* = 6.0 × 10^–18^] and with condition [*F*(1, 20) = 10.2, *p* = 4.5 × 10^–3^]. Differently from the analysis in the 0.1–30 Hz band, a significant interaction was also measured between segment duration and condition [*F*(3, 60) = 48.4, *p* = 3.6 × 10^–10^]. A three-way repeated measures ANOVA on the pitch decoding correlation metric indicated significant effects of method [*F*(1, 20) = 17.8, *p* = 4.2 × 10^–4^], segment duration [*F*(3, 20) = 157.0, *p* = 5.4 × 10^–15^], and condition were found [*F*(1, 20) = 33.9, *p* = 1.1 × 10^–5^]. As for the note-onset metric, significant interactions were shown for the pitch metric between decoding method and segment duration [*F*(3, 60) = 33.0, *p* = 6.4 × 10^–11^], between method and condition [*F* (1,20) = 7.6, *p* = 0.01], and between segment duration and condition [*F*(3, 60) = 24.0, *p* = 1.6 × 10^–7^].

**FIGURE 3:**
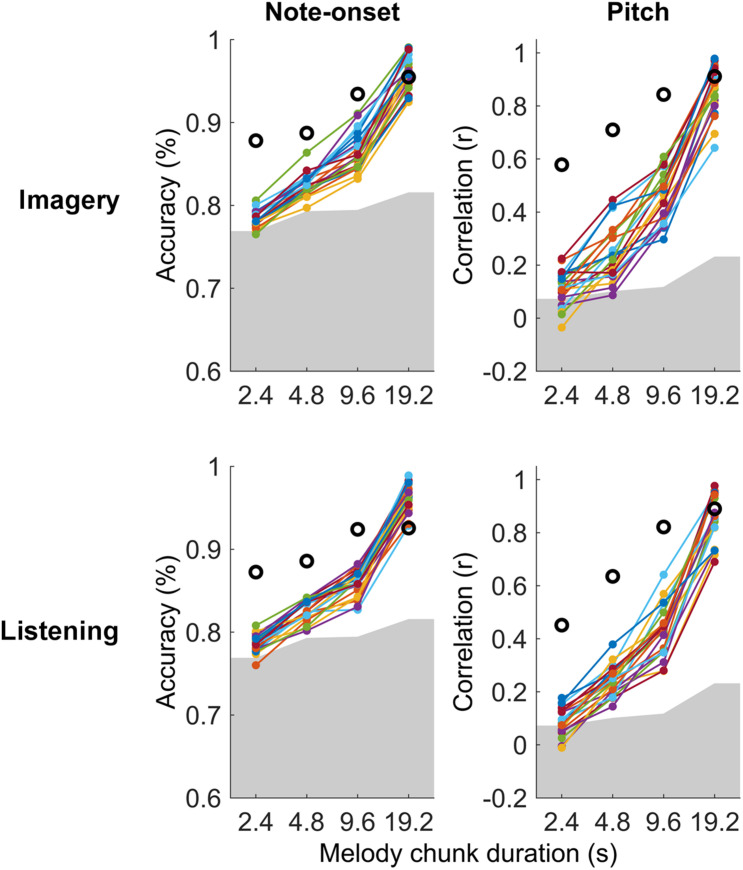
Single-subject melody decoding from EEG. Single-subject melody decoding from EEG (0.1–30 Hz) with maxCorr. Note-onset decoding accuracies (left) and pitch decoding correlations (right) are reported for the imagery and listening conditions. Grey shaded areas indicate the chance level (95th percentile of a distribution obtained by shuffling 100 times the identification indices of the EEG segments for each subject). Black circles indicate the decoding for the virtual best subject obtained by extracting EEG signal components that are most consistent across all participants with MCCA. Supplementary Figure 2 shows the results of this analysis for EEG filtered in the frequency band 1–30 Hz.

Significant melody decoding were also measured at the individual subject level with maxCorr (Figure 3). Specifically, all participants showed significant note-onset decoding scores for segment with a duration greater or equal to 4.8 s, in both imagery and listening conditions (permutation test, *N* = 100). Significant decoding was also measured for the shortest segment length (2.4 s) in 16 and 18 out of 21 or listening and imagery conditions, respectively. While this was unsurprising as the EEG signal is an excellent means to measure the timing of sensory responses, significance at the individual subject level was also measured for pitch decoding across all segment durations for 20 out of 21 participants.

The individual-subject result was then compared with the decoding scores obtained when considering the virtual best subject, obtained by extracting a signal that is most consistent across all participants with MCCA. This virtual best subject has in principle a higher SNR than any individual participant, thus providing decoding scores that should be at least as high as the best participant. Note that the MCCA procedure relies on the assumption that there exist some level of consistency in the EEG responses recorded from the 21 participants. The result in Figure 3 indicates that this was the case in both the listening and imagery conditions. In fact, the measured decoding scores for the virtual subject were larger than those for any individual participant.

**FIGURE 4:**
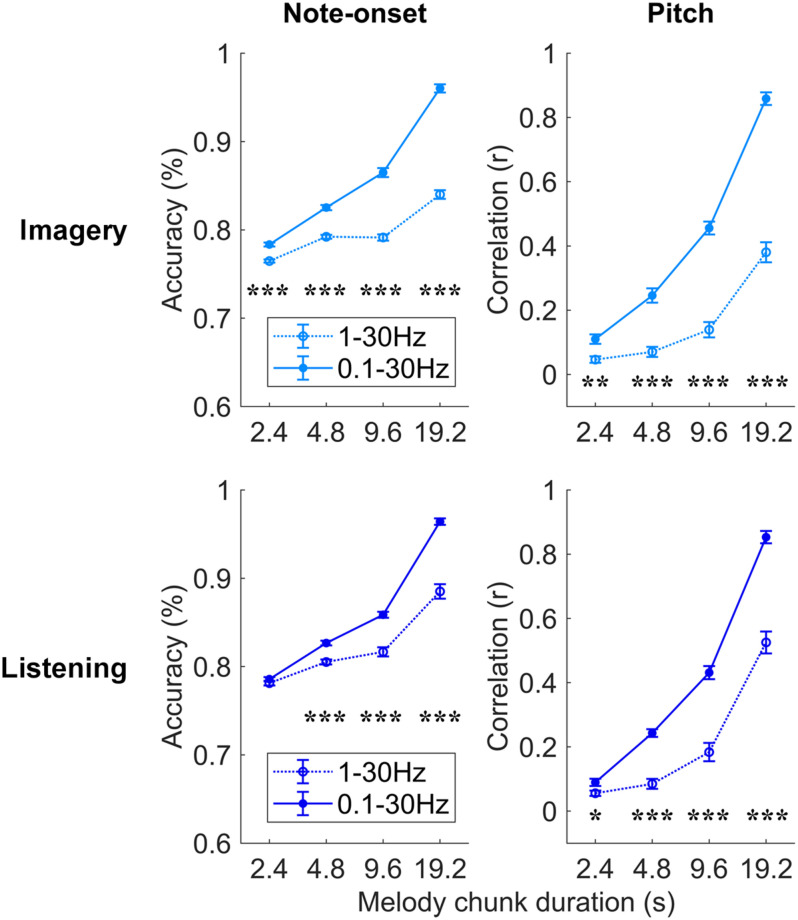
Robust melody decoding relies on low-frequency EEG (<1 Hz). Melody decoding scores obtained with maxCorr are compared for EEG filtered in the bands 0.1–30 Hz and 1–30 Hz. The inclusion of the low frequencies between 0.1 and 1 Hz largely increases the decoding scores in the imagery and listening conditions (three-way repeated measures ANOVA *p* < 0.05, *post hoc* Tukey’s HSD; *p < 0.05, **p < 0.01, ***p < 0.001). Supplementary Figure 3 shows the results of this analysis for the bTRF_*env*_ decoding method.

Further analyses were conducted to assess which EEG frequencies were most relevant for melody encoding during music imagery and listening (Figure 4). To this end, the impact of low delta frequencies (<1 Hz), which have been shown to encode melodic expectations, was assessed by comparing the decoding results for EEG filtered between 0.1–30 and 1–30 Hz directly. A three-way repeated measures ANOVA on the note-onset accuracy metric indicated significant effects of: frequency-band [0.1–30 Hz vs. 1–30 Hz; *F*(1, 20) = 369.8, *p* = 2.3 × 10^–14^]; condition [imagery vs. listening; *F*(1, 20) = 15.5, *p* = 8.1 × 10^–4^]; and segment duration [*F*(3, 60) = 1675.8, *p* = 1.0 × 10^–36^]. Significant interactions were measured between frequency-band and segment duration [*F*(3, 60) = 165.3, *p* = 3.3 × 10^–20^], between frequency-band and condition [*F*(1, 20) = 39.1, *p* = 4.2 × 10^–6^], and between segment duration and condition [*F*(3, 60) = 9.1, *p* = 1.1 × 10^–3^]. The same three-way repeated measures ANOVA procedure was used on the pitch correlation metric, indicating significant effects of: frequency-band [*F*(1, 20) = 201.6, *p* = 6.6 × 10^–12^] and segment duration [*F*(3, 60) = 757.4, *p* = 1.6 × 10^–32^], but no significant effect of condition [*F*(1, 20) = 2.7, *p* = 0.12]. Significant interactions were measured between frequency-band and segment duration [*F*(3, 60) = 85.8, *p* = 1.7 × 10^–17^], between frequency-band and condition [*F*(1, 20) = 9.9, *p* = 5.1 × 10^–3^], and between segment duration and condition [*F*(3, 60) = 5.9, *p* = 6.2 × 10^–3^]. *Post hoc* Tukey’s HSD indicated that low-frequency EEG < 1 Hz greatly contributes to the decoding of melodies in both the imagery and listening conditions. The individual-subject results for the band 1–30 Hz are reported in Supplementary Figure 2. Note that, differently from the band 0.1–30 Hz, the MCCA analysis on the 1–30 Hz EEG led to decoding scores that are larger than most participants in the listening condition only, while the imagery MCCA showed lower decoding scores than most individual participants. This could indicate that the 1–30 Hz EEG is substantially variable across participants in the imagery condition, causing difficulty in extracting common patterns for the accurate decoding of melodies. Another possible cause of this result could be a low SNR of imagery neural activity in the 1–30 Hz band for the imagery condition.

The use of a broader EEG frequency-band provides us with additional information that, when relevant to melody decoding, can improve the quality of the decoded melody. Indeed, the improved decoding also depends on the ability of the decoding model itself to learn and take advantage of such additional information. The result in Figure 4 demonstrates that low EEG frequencies between 0.1 and 1 Hz provides us with information that was not present in the faster frequencies, and that maxCorr is sensitive to such slow melody-related information. Interestingly the role of such low frequencies was not as prominent and even absent in the case of bTRF_*env*_. This result is detailed in Supplementary Figure 3, which shows that the inclusion of low-frequency EEG rhythms (0.1–1 Hz) in the decoding analysis improves the results for imagery but not for listening. Specifically, a three-way repeated measures ANOVA on the note-onset accuracy metric indicated no significant main effect of frequency-band [0.1–30 Hz vs. 1–30 Hz; *F*(1, 20) = 3.2, *p* = 0.09]. Significant effects were instead measured for condition [imagery vs. listening; *F*(1, 20) = 48.8, *p* = 8.9 × 10^–7^]; and segment duration [*F*(3, 60) = 6.9, *p* = 0.01]. A significant main effect of frequency-band was instead measured with a three-way repeated measures ANOVA on the pitch correlation metric [*F*(1, 20) = 10.3, *p* = 4.4 × 10^–3^]. Significant effects were also measured for condition [*F*(1, 20) = 28.6, *p* = 3.1 × 10^–5^]; and segment duration [*F*(3, 60) = 56.6, *p* = 8.6 × 10^–12^].

## Discussion

This study demonstrates that melodies can be accurately decoded from EEG responses to music listening and imagery at the individual participant and trial level. The use of music stimuli with the same tempo ensured that the decoding was driven by the neural encoding of melody at the level of individual notes, rather than by overall differences in timing or tempo. In doing so, we provide novel insights into the neural underpinnings of listening and auditory imagery, demonstrating that low-frequency EEG signals robustly encode melodic properties beyond the sound envelope. Furthermore, our results indicate a functional distinction between low-frequency neural activity that is either slower or faster than 1 Hz, with slower neural signals greatly contributing to the melody decoding during both imagery and listening. Finally, the accurate results achieved with our relatively simple methodology open new opportunities for applied research that we discuss in this section.

Recent research on speech perception provided substantial evidence indicating that low-frequency neural signals in the delta- and theta-bands encode both acoustic features, such as the sound envelope and higher-level linguistic properties (Lalor and Foxe, 2010; Ding et al., 2014; Di Liberto et al., 2015, 2021b; Brodbeck et al., 2018; Alday, 2019; Obleser and Kayser, 2019). This multifaceted encoding has also been measured in the context of music, showing that non-invasive neural recordings reflect properties such as tonal structure (Koelsch and Friederici, 2003; Koelsch and Siebel, 2005; Sankaran et al., 2018), beat (Tal et al., 2017), and melodic expectations (Omigie et al., 2013; Di Liberto et al., 2020). Our study indicates that such a multifaceted neural encoding also occurs in the case of auditory imagery. In line with our recent finding that both music listening and imagery engage neural expectation processes (Di Liberto et al., 2021a; Marion et al., 2021), the present study demonstrates that EEG signals recorded during music listening and imagery encode information beyond the acoustic envelope, allowing for the decoding of short music segments (Figure 2). Pitch-related information emerged in both the 0.1–30 Hz and 1–30 Hz bands, with a large portion of that information being present in the low-frequency neural signals below 1 Hz (Figure 4). Interestingly, such slow rhythms below 1 Hz did not contribute to the note-onset decoding accuracy when using the linear sound envelope decoder bTRF_*env*_ (Supplementary Figure 3), indicating that such a decoding approach could not reliably capture valuable low-frequency EEG signals. One possibility is that such signals reflect music properties that are different from the acoustic envelope and, as such, are not explicitly described by the bTRF_*env*_.

One open issue is determining what specific music properties are encoded in the EEG signal and contribute to the decoding. Indeed, a robust EEG signature of absolute or relative pitch would explain the strong pitch decoding scores measured in the present study. However, that is not the only possible explanation. For example, the use of a limited set of music stimuli could lead to segments whose timing is unique within that dataset, especially when using stimuli with different tempo or when considering long segments of music. In those cases, of course, note timing information would be sufficient to correctly identify the segments. Here, we controlled for that issue by presenting stimuli with the same tempo and by including a pitch permutation baseline (Figure 2 and Supplementary Figure 1). The results showed that, although the decoding of longer segments (9.6 and 19.2 s) was solely driven by note timing information, neural signals reflecting more than note timing contributed to the melody decoding for the shorter segments (2.4 and 4.8 s; Figure 2, pitch correlation metric). Our results demonstrates that both timing and pitch-related EEG signals can contribute to melody decoding and, crucially, that the stimulus reconstructions obtained with envelope linear decoders can be substantially improved by using models capturing information beyond timing with methods such as maxCorr. We speculate that our decoding results may reflect the EEG encoding of a combination of pitch and other pitch-related signals (e.g., melodic context), which act here as proxy indices for pitch. Indeed, EEG has been shown to encode other pitch-related properties, such as the pitch expectation strengths (Omigie et al., 2013; Di Liberto et al., 2020), which could at least partly explain our results. Further work is needed to disentangle the factors contributing to the melody decoding, for example by using richer sets stimuli where the impact of pitch-related signals could be controlled and minimised.

The neural encoding of music was previously shown to be different during listening and imagery largely due to the presence and absence of an auditory stimulus, respectively (Di Liberto et al., 2021a). In fact, previous work showed both shared and non-overlapping neural activation during listening and imagery tasks, largely by means of high spatial-resolution functional recordings with fMRI (see Zatorre and Halpern, 2005 for a review). Interestingly, despite these dissimilarities, accurate melody decoding was possible in both conditions (Figure 3), indicating that both listened and imagined melodies elicit neural responses that are consistent across time and repetition. This consistency is of great importance as it confirms that studying imagery responses with methodologies that summarise neural signals across time, such as encoding models, is a valid and feasible approach. Indeed, this result is highly dependent on the use of an experimental paradigm allowing for a precise synchronisation of the imagery task, which was possible with naturalistic music here but may be more challenging for other stimuli, such as speech, whose precise synchronisation may require some loss in terms of ecological validity. Furthermore, the consistency in the neural responses to music measured in this study supports the hypothesis that a same music segment remains relevant for a listener across repetitions, eliciting a consistent neural activation at each occurrence. Extensive work and discussions have been conducted on this topic, however there remains considerable uncertainty on the precise impact of repetition in auditory perception (Margulis, 2014). One argument is that repetition has a special and fundamental role in music, while that is not the case in other auditory stimuli such as speech. For this reason, the central role of repetition in music may be at the basis of the remarkably consistent neural responses to listened and imagined music segments allowing for the accurate decoding of melodies.

Our results show that accurate decoding of melodies at the level of individual musical units can be achieved with a relatively simple procedure based on a maximum correlation metric. As such, the decoding scores in this study are certainly expected to improve by using more elaborate decoding procedures. This possibility encourages further investigations on BCIs based on imagery tasks. For example, the present work may constitute a starting point for the development of BCI solutions for the rapid selection of songs and other audio material in individuals with mobility impairment. In fact, the decoding results in the musical imagery condition were significant on most individual participants and for segments as short as 2.4 s. Note that the MCCA and JD procedures indicated consistent neural responses across time and participants both within the listening and imagery conditions, which is an encouraging result for future BCI solutions. Furthermore, the ability to decode melodies at the level of individual musical units offer a new opportunity for the continuous monitoring of cognitive functions that have a central role in music perception, such as attention and prediction.

Our finding is particularly relevant for BCI applications as the decoding method, organised into reference and test sets, could be promptly applied in realistic settings involving the decoding of melodies that were previously presented to the user. In that context, the decoding results for different segment durations inform us on how rapidly and accurately a song could be identified. It should be noted that the use of music pieces with different tempo would likely improve our decoding scores, thus making a BCI even more rapid and accurate. However, that was not the focus of this investigation as the paradigm was optimised to investigate the multifaceted contributors to music perception while controlling for the dominant encoding of timing. Future studies should also explore the possibility of extending the paradigm to decode unseen songs, for which there would be no prior EEG data available, which could be possible in the presence of a rich reference EEG set. In this regard, further work is needed to determine optimal reference sets with sufficient musical variability to generalise and allow for the accurate decoding of unseen songs within a given musical culture or genre. Another open challenge is to determine if the present findings apply to the general population. The choice of focusing on expert trained musicians was driven by experimental and not theoretical reasons. In fact, the imagery task used here requires some level of training to be performed accurately and reliably. It could be possible to utilise this experimental paradigm on non-musicians too. While we speculate that our results apply to the general population, note that we would expect weaker decoding scores in the general population due to reduced and variable (over time and participants) task performance, making it difficult to use that decoding analysis to assess differences in the neural representation of imagined and listened music itself. Other tasks less reliant on individual imagery temporal precision could provide us with a more fair comparison between trained musicians and the general population, for example paradigms involving imagery during silent gaps in familiar music (e.g., Gabriel et al., 2016).

## Data Availability Statement

Publicly available datasets were analyzed in this study. This data can be found here: https://datadryad.org/stash/dataset/doi:10.5061/dryad.dbrv15f0j. Further inquiries can be directed to the corresponding author/s.

## Ethics Statement

The study was undertaken in accordance with the Declaration of Helsinki and was approved by the CERES Committee of Paris Descartes University (CERES 2013-86 11). The patients/participants provided their written informed consent to participate in this study.

## Author Contributions

GD and SS conceived the study. GM and GD designed the experiment. GM run the experiment. GD analysed the data and wrote the first draft of the manuscript. SS and GM edited the manuscript. EEG experiments were part of previous studies by the authors. All authors contributed to the article and approved the submitted version.

## Conflict of Interest

The authors declare that the research was conducted in the absence of any commercial or financial relationships that could be construed as a potential conflict of interest.

## Publisher’s Note

All claims expressed in this article are solely those of the authors and do not necessarily represent those of their affiliated organizations, or those of the publisher, the editors and the reviewers. Any product that may be evaluated in this article, or claim that may be made by its manufacturer, is not guaranteed or endorsed by the publisher.

## Footnotes

1
https://giamusicassessment.com/

## Supplementary Material

Supplementary Figure 1 Note and pitch decoding (1–30 Hz EEG). Melody decoding scores obtained with maxCorr from EEG data filtered between 1 and 30 Hz are compared with scores obtained when using backward envelope TRFs (bTRF_*env*_) for the imagery **(A)** and listening **(B)** conditions. Decoding accuracies are reported for note timing (left) and decoding correlation values for note pitch values (right). A three-way repeated measures ANOVA indicated a significant effect of decoding method (note-onset metric: *p* = 0.03; pitch metric: *p* = 4.2 × 10^–4^; *post hoc* Tukey’s HSD; **p* < 0.05, ***p* < 0.01, ****p* < 0.001). Grey lines indicate the pitch decoding correlation scores with the maxCorr method when pitch information was shuffled among segments with identical timing. Scores larger than this baseline indicate that the decoding is partially driven by pitch-related EEG information. Bottom panels indicate the effect size (Cohen’s d) of the comparison between maxCorr and bTRFenv (blue dotted lines) and between maxCorr and the maxCorr baseline after shuffling the pitch values (grey solid lines).

Supplementary Figure 2 Single-subject Melody Decoding (1–30 Hz EEG). Single-subject melody decoding from EEG (1–30 Hz) with maxCorr. Note-onset decoding accuracies (left) and pitch decoding correlations (right) are reported for the imagery and listening conditions. Grey shaded areas indicate the chance level (95th percentile of a distribution obtained by shuffling 100 times the identification indices of the EEG segments for each subject).

Supplementary Figure 3 Linear decoding models (bTRF_*env*_) fail to capture low-frequency EEG (< 1 Hz) melody information in the listening condition. Melody decoding scores obtained with bTRF_*env*_ are compared for EEG filtered in the bands 0.1–30 Hz and 1–30 Hz. The inclusion of the low frequencies between 0.1 and 1 Hz largely increases the decoding scores in the imagery and listening conditions (three-way repeated measures ANOVA, main effect of frequency-band; Note-onset: *p* = 0.09; pitch: *p* = 4.4 × 10^–3^, *post hoc* Tukey’s HSD; **p* < 0.05, ***p* < 0.01, ****p* < 0.001).

## References

[B1] Alday P. M. (2019). M/EEG analysis of naturalistic stories: a review from speech to language processing. *Lang. Cogn. Neurosci.* 34 457–473. 10.1080/23273798.2018.1546882

[B2] Brodbeck C. Hong L. E. Simon J. Z. (2018). Rapid transformation from auditory to linguistic representations of continuous speech. *Curr. Biol.* 28 3976–3983.e5. 30503620 10.1016/j.cub.2018.10.042 PMC6339854

[B3] Carrus E. Pearce M. T. Bhattacharya J. (2013). Melodic pitch expectation interacts with neural responses to syntactic but not semantic violations. *Cortex* 49 2186–2200. 10.1016/J.CORTEX.2012.08.024 23141867

[B4] Crosse M. J. Di Liberto G. M. Bednar A. Lalor E. C. (2016). The multivariate temporal response function (mTRF) toolbox: a MATLAB toolbox for relating neural signals to continuous stimuli. *Front. Hum. Neurosci.* 10:604. 10.3389/fnhum.2016.00604 27965557 PMC5127806

[B5] de Cheveigné A. Di Liberto G. M. Arzounian D. Wong D. Hjortkjaer J. Fuglsang S. A. (2019). Multiway canonical correlation analysis of brain data. *bioRxiv.* 10.1016/j.neuroimage.2018.11.026 30496819

[B6] de Cheveigné A. Parra L. C. (2014). *Joint Decorrelation, a Versatile Tool for Multichannel Data Analysis.* Available online at: http://www.sciencedirect.com/science/article/pii/S1053811914004534 (accessed December 30, 2017). 10.1016/j.neuroimage.2014.05.068 24990357

[B7] de Cheveigné A. Simon J. Z. (2008). Denoising based on spatial filtering. *J. Neurosci. Methods* 171 331–339. 10.1016/j.jneumeth.2008.03.015 18471892 PMC2483698

[B8] de Cheveigné A. Wong D. E. Di Liberto G. M. Hjortkjær J. Slaney M. Lalor E. (2018). Decoding the auditory brain with canonical component analysis. *Neuroimage* 172 206–216. 10.1016/j.neuroimage.2018.01.033 29378317

[B9] Di Liberto G. M. Marion G. Shamma S. A. (2021a). The music of silence. Part II: musical listening induces imagery responses. *J. Neurosci, JN-RM-0184-21R2*. 10.1523/JNEUROSCI.0184-21.2021 34341154 PMC8412992

[B10] Di Liberto G. M. Nie J. Yeaton J. Khalighinejad B. Shamma S. A. Mesgarani N. (2021b). Neural representation of linguistic feature hierarchy reflects second-language proficiency. *Neuroimage* 227:117586. 10.1016/j.neuroimage.2020.117586 33346131 PMC8527895

[B11] Di Liberto G. M. O’Sullivan J. A. Lalor E. C. (2015). Low-frequency cortical entrainment to speech reflects phoneme-level processing. *Curr. Biol.* 25 2457–2465. 10.1016/j.cub.2015.08.030 26412129

[B12] Di Liberto G. M. Pelofi C. Bianco R. Patel P. Menhta A. D. Herrero J. L. (2020). Cortical encoding of melodic expectations in human temporal cortex. *eLife* 9:e51784. 10.7554/eLife.51784 32122465 PMC7053998

[B13] Ding N. Chatterjee M. Simon J. Z. (2014). Robust cortical entrainment to the speech envelope relies on the spectro-temporal fine structure. *Neuroimage* 88 41–46. 24188816 10.1016/j.neuroimage.2013.10.054 PMC4222995

[B14] Freitas C. Manzato E. Burini A. Taylor M. J. Lerch J. P. Anagnostou E. (2018). Neural correlates of familiarity in music listening: a systematic review and a neuroimaging meta-analysis. *Front. Neurosci.* 12:686. 10.3389/fnins.2018.00686 30344470 PMC6183416

[B15] Gabriel D. Wong T. C. Nicolier M. Giustiniani J. Mignot C. Noiret N. (2016). Don’t forget the lyrics! Spatiotemporal dynamics of neural mechanisms spontaneously evoked by gaps of silence in familiar and newly learned songs. *Neurobiol. Learn. Mem.* 132 18–28. 10.1016/j.nlm.2016.04.011 27131744

[B16] Hotelling H. (1936). Relations between two sets of variates. *Biometrika* 28 321–377. 10.2307/2333955

[B17] Jagiello R. Pomper U. Yoneya M. Zhao S. Chait M. (2019). Rapid brain responses to familiar vs. unfamiliar music – an EEG and Pupillometry study. *Sci. Rep.* 9:15570. 10.1038/s41598-019-51759-9 PMC6821741 31666553

[B18] Koelsch S. (2011). Toward a neural basis of music perception – A review and updated model. *Front. Psychol.* 2:110. 10.3389/fpsyg.2011.00110 21713060 PMC3114071

[B19] Koelsch S. Friederici A. D. (2003). “Toward the neural basis of processing structure in music. Comparative results of different neurophysiological investigation methods. *Ann. N. Y. Acad. Sci.* 999 15–28. 10.1196/annals.1284.002 14681114

[B20] Koelsch S. Siebel W. A. (2005). Towards a neural basis of music perception. *Trends Cogn. Sci.* 9 578–584. 10.1016/J.TICS.2005.10.001 16271503

[B21] Lalor E. C. Foxe J. J. (2010). Neural responses to uninterrupted natural speech can be extracted with precise temporal resolution. *Eur. J. Neurosci.* 31 189–193. 10.1111/j.1460-9568.2009.07055.x 20092565

[B22] Lalor E. C. Power A. J. Reilly R. B. Foxe J. J. (2009). Resolving precise temporal processing properties of the auditory system using continuous stimuli. *J. Neurophysiol.* 102 349–359. 10.1152/jn.90896.2008 19439675

[B23] Margulis E. H. (2014). *On Repeat.* Oxford: Oxford University Press. 10.1093/acprof:oso/9780199990825.001.0001

[B24] Marion G. Di Liberto G. M. Shamma S. A. (2021). The music of silence. Part I: responses to musical imagery accurately encode melodic expectations and acoustics. *J Neurosci, JN-RM-0183-21R3.*. 10.1523/JNEUROSCI.0183-21.2021 34341155 PMC8412990

[B25] Obleser J. Kayser C. (2019). Neural Entrainment and Attentional Selection in the Listening Brain. *Trends Cogn. Sci.* 23 913–926. 10.1016/j.tics.2019.08.004 31606386

[B26] Omigie D. Pearce M. T. Williamson V. J. Stewart L. (2013). Electrophysiological correlates of melodic processing in congenital amusia. *Neuropsychologia* 51 1749–1762. 10.1016/j.neuropsychologia.2013.05.010 23707539

[B27] Patel A. D. (2003). “A New Approach to the Cognitive Neuroscience of Melody,” in *The Cognitive Neuroscience of Music*, eds Peretz I. Zatorre R. (Oxford: Oxford University Press). 10.1093/acprof:oso/9780198525202.003.0021

[B28] Sankaran N. Thompson W. F. Carlile S. Carlson T. A. (2018). Decoding the dynamic representation of musical pitch from human brain activity. *Sci. Rep.* 8:839. 10.1038/s41598-018-19222-3 PMC5770452 29339790

[B29] Tal I. Large E. W. Rabinovitch E. Wei Y. Schroeder C. E. Poeppel D. (2017). Neural entrainment to the beat: the “missing-pulse” phenomenon. *J. Neurosci.* 37 6331–6341. 10.1523/JNEUROSCI.2500-16.2017 28559379 PMC5490067

[B30] Zatorre R. J. Halpern A. R. (2005). Mental concerts: musical imagery and auditory cortex. *Neuron* 47 9–12. 10.1016/j.neuron.2005.06.013 15996544

