# Supplementary figures and images for: Accurate Decoding of Imagined and Heard Melodies
Source: Front Neurosci. 2021 Aug 5;15:673401. doi: 10.3389/fnins.2021.673401 (PMC8375770; doi:10.3389/fnins.2021.673401)

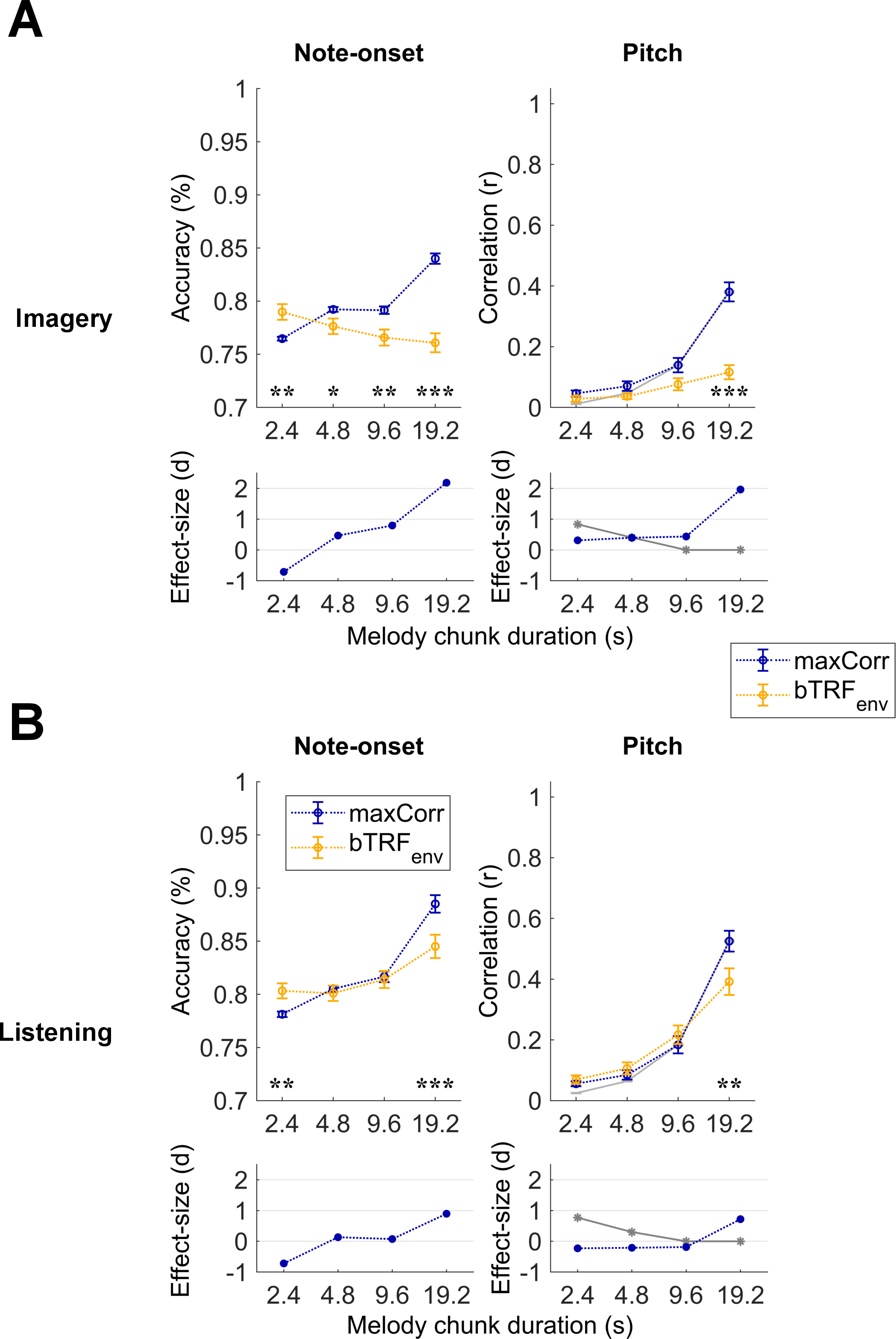

Supplement: Supplementary Figure 1 — Note and pitch decoding (1–30 Hz EEG). Melody decoding scores obtained with maxCorr from EEG data filtered between 1 and 30 Hz are compared with scores obtained when using backward envelope TRFs (bTRFenv) for the imagery (A) and listening (B) conditions. Decoding accuracies are reported for note timing (left) and decoding correlation values for note pitch values (right). A three-way repeated measures ANOVA indicated a significant effect of decoding method (note-onset metric: p = 0.03; pitch metric: p = 4.2 × 10^–4^; post hoc Tukey’s HSD; *p < 0.05, **p < 0.01, ***p < 0.001). Grey lines indicate the pitch decoding correlation scores with the maxCorr method when pitch information was shuffled among segments with identical timing. Scores larger than this baseline indicate that the decoding is partially driven by pitch-related EEG information. Bottom panels indicate the effect size (Cohen’s d) of the comparison between maxCorr and bTRFenv (blue dotted lines) and between maxCorr and the maxCorr baseline after shuffling the pitch values (grey solid lines). [file Image_1.TIF]

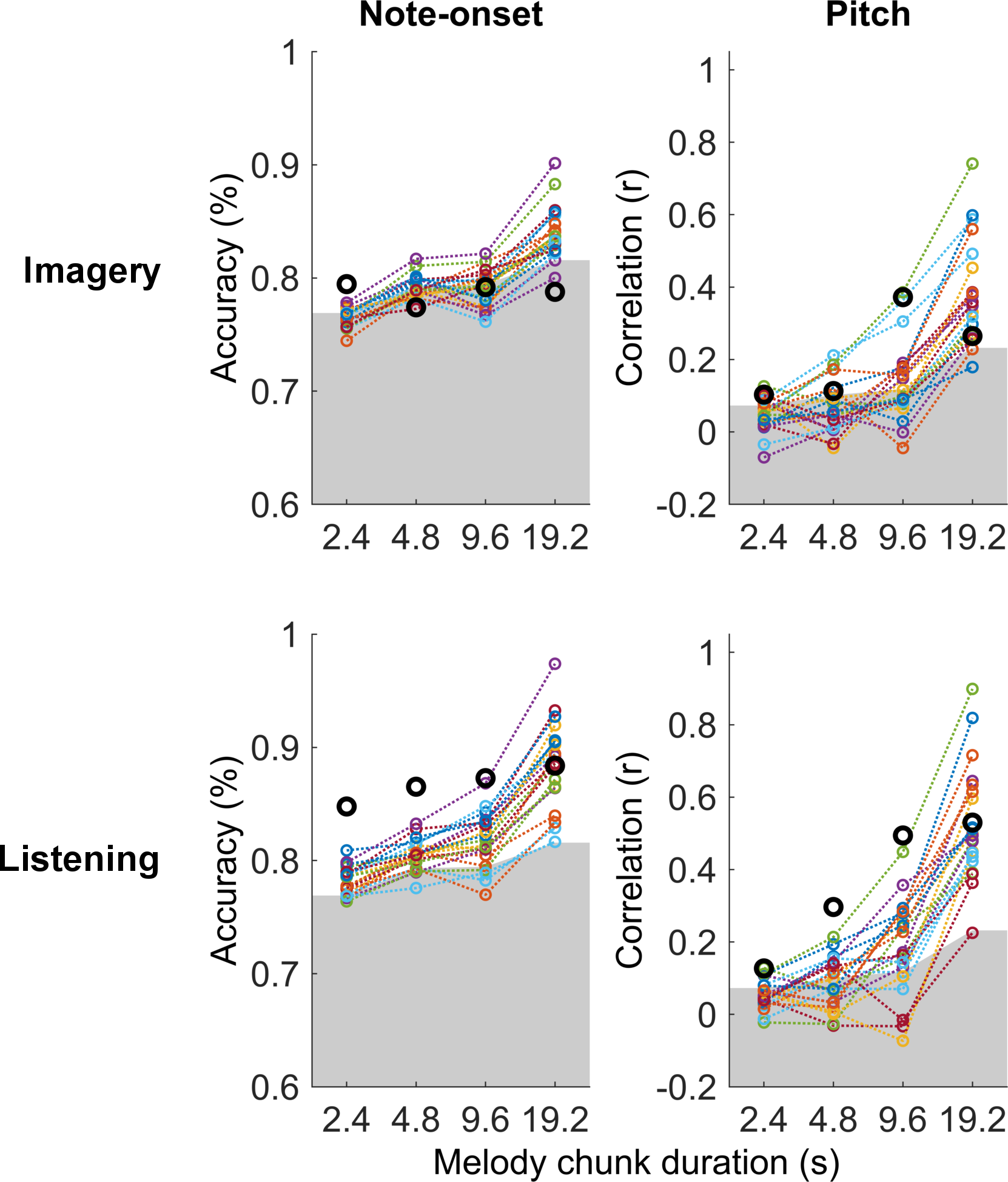

Supplement: Supplementary Figure 2 — Single-subject Melody Decoding (1–30 Hz EEG). Single-subject melody decoding from EEG (1–30 Hz) with maxCorr. Note-onset decoding accuracies (left) and pitch decoding correlations (right) are reported for the imagery and listening conditions. Grey shaded areas indicate the chance level (95th percentile of a distribution obtained by shuffling 100 times the identification indices of the EEG segments for each subject). [file Image_2.TIF]

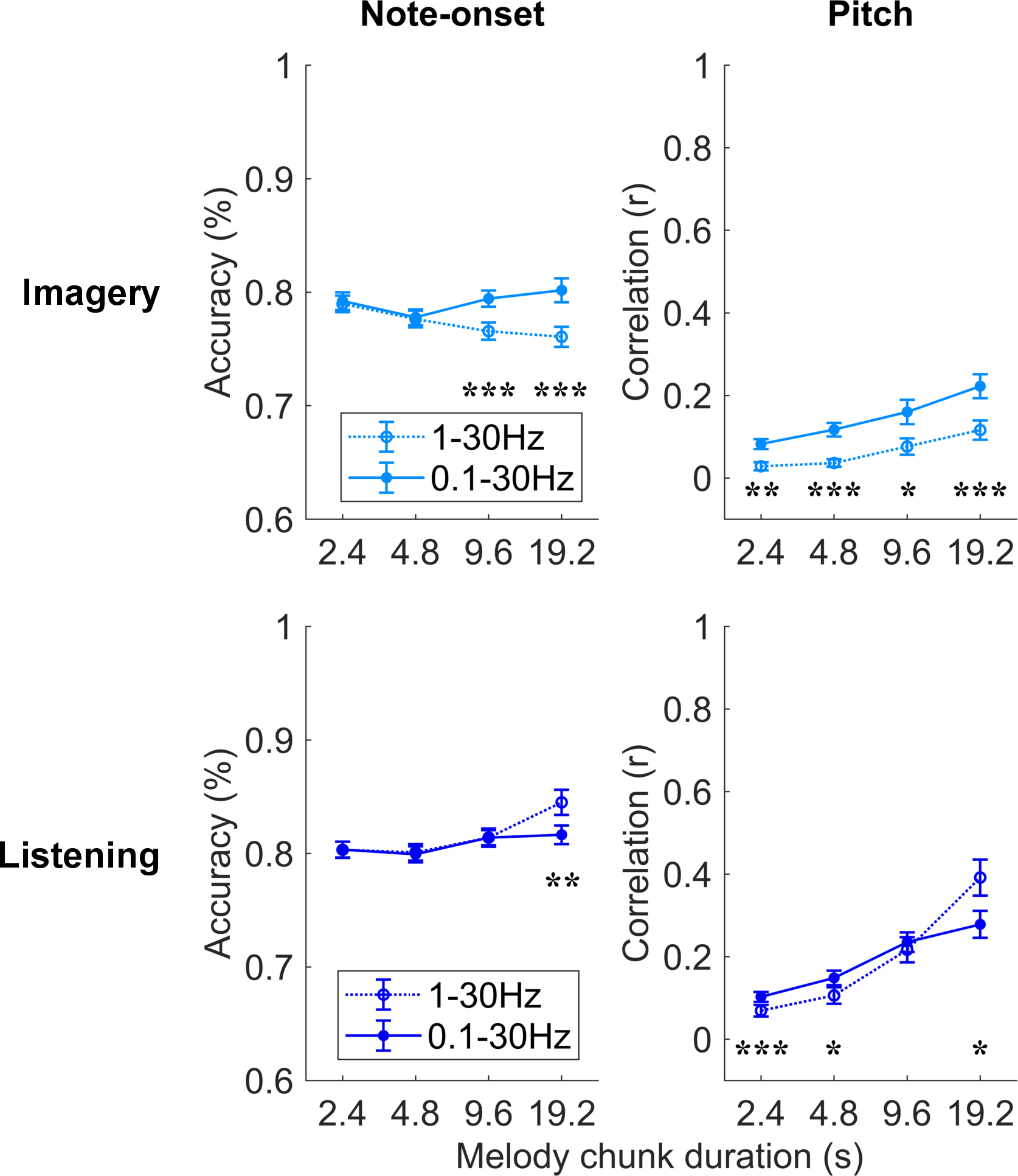

Supplement: Supplementary Figure 3 — Linear decoding models (bTRFenv) fail to capture low-frequency EEG (< 1 Hz) melody information in the listening condition. Melody decoding scores obtained with bTRFenv are compared for EEG filtered in the bands 0.1–30 Hz and 1–30 Hz. The inclusion of the low frequencies between 0.1 and 1 Hz largely increases the decoding scores in the imagery and listening conditions (three-way repeated measures ANOVA, main effect of frequency-band; Note-onset: p = 0.09; pitch: p = 4.4 × 10^–3^, post hoc Tukey’s HSD; *p < 0.05, **p < 0.01, ***p < 0.001). [file Image_3.TIF]
